# A prospective clinical study of the influence of oral protein intake on [^18^F]FET-PET uptake and test–retest repeatability in glioma

**DOI:** 10.1186/s13550-024-01119-0

**Published:** 2024-06-26

**Authors:** Sarah Chehri, Otto Mølby Henriksen, Lisbeth Marner, Mette Christensen, Aida Muhic, Hans Skovgaard Poulsen, Ian Law

**Affiliations:** 1 Department of Clinical Physiology and Nuclear Medicine, Copenhagen University Hospital - Rigshospitalet, Blegdamsvej 9, 2100 Copenhagen, Denmark; 2 Department of Radiation Biology, Copenhagen University Hospital - Rigshospitalet, Blegdamsvej 9, 2100 Copenhagen, Denmark; 3 Department of Clinical Physiology and Nuclear Medicine, Copenhagen University Hospital Bispebjerg, Bispebjerg Bakke 23, 2400 Copenhagen, Denmark; 4 Department of Clinical Medicine, University of Copenhagen, Copenhagen, Denmark; 5 Department of Clinical Genetics, Copenhagen University Hospital - Rigshospitalet, Blegdamsvej 9, 2100 Copenhagen, Denmark; 6 Department of Oncology, Copenhagen University Hospital - Rigshospitalet, Blegdamsvej 9, 2100 Copenhagen, Denmark

**Keywords:** Glioma, Amino acid positron emission tomography, Test–retest, Brain tumour, Neuro-oncology

## Abstract

**Background:**

O-(2-[^18^F]fluoroethyl)-l-tyrosine positron emission tomography ([^18^F]FET PET) scanning is used in routine clinical management and evaluation of gliomas with a recommended 4 h prior fasting. Knowledge of test–retest variation of [^18^F]FET PET imaging uptake metrics and the impact of accidental protein intake can be critical for interpretation. The aim of this study was to investigate the repeatability of [^18^F]FET-PET metrics and to assess the impact of protein-intake prior to [^18^F]FET PET scanning of gliomas.

**Results:**

Test–retest variability in the non-protein group was good with absolute (and relative) upper and lower limits of agreement of + 0.15 and − 0.13 (+ 9.7% and − 9.0%) for mean tumour-to-background ratio (TBR_mean_), + 0.43 and − 0.28 (+ 19.6% and − 11.8%) for maximal tumour-to-background ratio (TBR_max_), and + 2.14 cm^3^ and − 1.53 ml (+ 219.8% and − 57.3%) for biological tumour volume (BTV). Variation was lower for uptake ratios than for BTV. Protein intake was associated with a 27% increase in the total sum of plasma concentration of the l-type amino acid transporter 1 (LAT1) relevant amino acids and with decreased standardized uptake value (SUV) in both healthy appearing background brain tissue (mean SUV − 25%) and in tumour (maximal SUV − 14%). Oral intake of 24 g of protein 1 h prior to injection of tracer tended to increase variability, but the effects on derived tumour metrics TBR_mean_ and TBR_max_ were only borderline significant, and changes generally within the variability observed in the group with no protein intake.

**Conclusion:**

The test–retest repeatability was found to be good, and better for TBR_max_ and TBR_mean_ than BTV, with the methodological limitation that tumour growth may have influenced results. Oral intake of 24 g of protein one hour before a [^18^F]FET PET scan decreases uptake of [^18^F]FET in both tumour and in healthy appearing brain, with no clinically significant difference on the most commonly used tumour metrics.

**Supplementary Information:**

The online version contains supplementary material available at 10.1186/s13550-024-01119-0.

## Background

The response assessment in neurooncology working group (PET RANO) recommends positron emission tomography (PET) scans for diagnosis, evaluation, clinical management and treatment assessment of gliomas [1, 2]. The radio-labelled amino acid analogue O-(2-[^18^F]fluoroethyl)-l-tyrosine ([^18^F]FET) as well as the analogues l-[^18^F]fluorodopa and [^11^C]methionine deliver exceptional tumour-to-background contrast and is used routinely for delineation of glioma extent in surgical and radiotherapy treatment planning [3, 4], for differentiation between tumour recurrence and treatment related effects, and for post-treatment surveillance [5–7].

The [^18^F]FET uptake in glioma cells is considered to be induced by an overexpression of the L-type amino acid transporter type 1 (LAT1). LAT1 is mainly responsible for the transport of branched-chain amino acids (AMA) i.e. valine (VAL), isoleucine (ILE), and leucine (LEU) as well as the neutral bulky amino acids; phenylalanine (PHE), tyrosine (TYR), tryptophane (TRP), histidine (HIS) and methionine (MET) [8, 9]. LAT1 is highly expressed in a variety of human cancer tissues and is correlated with the malignant phenotypes and proliferation of gliomas [10]. However, the influence of plasma amino acid levels on the uptake of [^18^F]FET in gliomas is complex for several reasons [11]. LAT1 works as an obligatory exchanger (antiport) by coupling the influx of an extracellular amino acid substrate to the efflux of an intracellular amino acid substrate across a membrane. It has been shown that [^18^F]FET competes with other LAT1 substrates for uptake in glioblastoma cells and that LAT1 is less effective in recognizing [^18^F]FET as an efflux substrate leading to a tumour-specific accumulation [12]. The prediction would thus be that increased concentration of LAT1-relevant AMAs in plasma prior to [^18^F]FET injection would decrease [^18^F]FET uptake in the brain, as have been found in a single healthy subject [13] and in patients with phenylketonuria (PKU) [14] using l-[^18^F]fluorodopa, and in brain tumour patients studied with l-3-[^123^I]iodo-alpha-methyl-l-tyrosine ([^123^I]IMT) single photon emission tomography (SPECT) [15]. However, studies of osteosarcoma cells have shown that [^18^F]FET uptake is increased after intracellular preload with LAT1-relevant AMA by increasing the intracellular LAT1 substrate [8]. Thus, oral protein intake prior to a PET scanning could paradoxically increase [^18^F]FET uptake in glioma. Furthermore, neoplastically transformed cells may synthesize atypical transport systems [16] that may unpredictably alter [^18^F]FET uptake. This could be one of the mechanisms underlying the variable or missing correlation between tissue LAT1 staining intensity and [^18^F]FET uptake [17, 18].

For any clinical biomarker in oncology, robustness and knowledge of the repeatability of the method is essential when assessing changes between different time points, e.g. response to therapies and detection of tumour progression or recurrence. In a clinical setting it is not uncommon that patients need rescheduling of their PET scans following food intake contrary to instruction, in order to avoid potential suppression of tumour uptake. This is unfortunate as important diagnostic information will be delayed, and resources lost. Knowledge of test–retest *variability* in humans is sparse [19] with very limited data of test–retest *repeatability* available for the most important clinical [^18^F]FET-PET tumour metrics or the influence of prior oral protein intake on these metrics. Understanding the impact of protein intake prior to a PET scan can potentially modify the requirement for fasting, which for many patients is challenging and associated with excessive discomfort, especially for children, individuals with diabetes and glioma patients with post-treatment cognitive deficits.

To address these issues related directly to clinical patient management and to aid the interpretation of [^18^F]FET PET scanning in diagnosed glioma patients undergoing clinical follow-up, we examined both the test–retest repeatability and the influence of oral protein-intake prior to scanning in glioma patients.

## Methods

### Study design and patients

The study was conducted as a prospective, clinical, single-centre, test–retest trial. The study was approved by the Scientific Ethics Committee of the Capital Region (H-1-2013-062). All patients gave written informed consent to participate in the study after oral and written information.

**Fig. 1:**
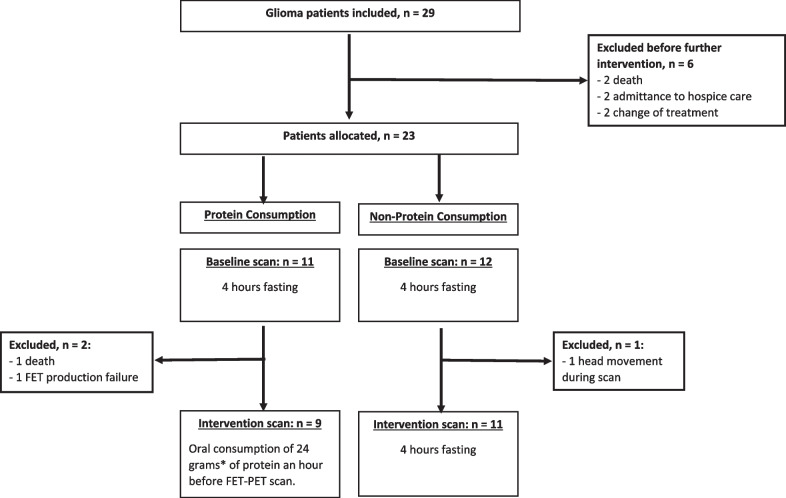
Inclusion flow diagram of the study population

Between April 2016 and May 2017, 29 glioma patients scheduled for a clinical [^18^F]FET PET examination signed informed consent for participation. All patients had histologically verified glioma [20] and were in their clinical follow-up period, after or in-between series of treatment. Exclusion criteria were; inability to undergo [^18^F]FET PET examination, or scheduled for surgery, radiation- or chemotherapy within 7 days after the first [^18^F]FET PET scan. Twenty-three participants were at random allocated by minimization to either the non-protein consumption (NP) group or the protein consumption (PC) group with the exception that patients unwilling to consume the protein drink were a priori allocated to the NP group. A total of six patients were excluded before group allocation and a further three patients were excluded between the first and the second scan day. Flow chart of inclusion is shown in Fig. 1.

The remaining 20 patients (Table 1) underwent two [^18^F]FET PET scans with an interval of maximum 7 days (3 days n = 8, 4 days n = 1, 7 days n = 11) between each scan and with no intervening treatment. Patients were instructed to consume neither protein nor food for at least 4 h prior to both scans. On the (intended) second scan day patients in the PC group were following fasting blood sampling given 250 ml of nutritional weight gain drink (Nutricia Nutridrink Compact^®^) containing 24 g of protein, equivalent to eating four large eggs or a can of tuna fish [21], to consume orally one hour before injection of [^18^F]FET. The first patient in the PC group consumed double volume (48 g of protein) to assess patient acceptability of protein load. However, this amount of protein was found too nauseating to consume and was reduced for the following patients. For two patients in the PC group, protein was provided on the first scan day due to non-compliance of fasting requirements. The PC protein consumption scan and the second PET scan from the NP group were designated as the “Intervention” scan, and the non-protein consumption scan of the PC group and the first scans of the NP group were designated “baseline” scans.

**Table 1:** Patient characteristics

Patient No.	Sex	Age (years)	Glioma type	MGMT status	Prior treatment Radio therapy/surgery	WHO PS	Steroid > 25 mg	Time from surgery/RT (months)	Progressive disease course
*PC group*									
1	M	46	GBM	+	STR, RT, BV/Lom	0	–	23	No
2	M	58	GBM	+	STR, RT, BV	1	–	16	No
3	F	37	A2	N/A	STR, RT	0	–	5	No
4	M	34	A3 unspec	–	RT, TMZ	0	–	58	No
5	M	42	OD3	+	STR × 6, RT, TMZ, PCV, BV	2	+	22	Yes
6	M	59	GBM	+	GTR, RT, TMZ	0	–	9	No
7	M	33	A2/A3	+	STR, TMZ	0	–	1	No
8	F	56	A3	+	STR, RT	0	–	12	No
9	F	51	A3	+	GTR, RT, TMZ	0	–	10	No
*NP group*									
10	M	65	GBM*	+	GTR, RT, BV/Ir	0	–	72	No
11	F	68	OD2	N/A	PCV, TMZ	0	–	–	No
12	F	24	GBM	–	STR × 2, RT, TMZ	0	–	4	No
13	M	45	GBM	+	STR × 2, RT, TMZ	0	–	10	No
14	M	70	GBM	+	STR, RT, TMZ	1	–	8	Yes
15	M	51	A3/GBM**	N/A	GTR, RT, TMZ	1	–	3	Yes
16	M	33	A4	+	GTR, RT, TMZ	0	–	5	No
17	F	49	OD2	+	RT, TMZ	0	–	4	No
18	M	65	GBM	+	GTR, RT, TMZ	0	–	12	No
19	M	71	GBM	+	RT, TMZ, BV	0	+	14	Mixed
20	M	61	GBM	–	STR, RT, TMZ	1	–	6	Mixed

F,  female, M, male, A2, WHO CNS grade 2 astrocytoma, IDH mutant, A3, WHO CNS grade 3 astrocytoma, IDH mutant, A4, WHO CNS grade 4 astrocytoma, IDH mutant, GBM, WHO CNS grade 4 glioblastoma, IDH wild type, OD2, WHO CNS grade 2 oligodendroglioma, IDH-mutant, 1p/19q co-deletion, MGMT, O^6^-methylguanine-DNA methyltransferase promotor methylated, GTR, gross total resection, PCV, procarbazine/lomustine/vincristine, RT, radio therapy, STR, subtotal resection, TMZ, temozolomide, WHO, world health organization, BV, bevacizumab, Ir, irinotecan, PS, performance status. * Primary surgery and treatment abroad, and pathology report not available. **Primary surgery abroad, astrocytoma WHO III without IDH mutations

### Pathology and clinical course

Tumour histology were originally defined according to the 2007 WHO classification [20] of brain tumours at surgery or biopsy performed prior to the inclusion in the study. All patients had received prior treatment. Table1 summarizes patient characteristics and demographics including the glioma type and O^6^-methylguanin-DNA-methyltransferase (MGMT) promoter methylation and isocitrate dehydrogenase (IDH1) mutation status. Tumours were retrospectively reclassified based on the 2021 WHO classification [22] except in one patient (# 4) in whom IDH status was not available. To assess if tumour growth between scan days may potentially have influenced results, the disease course at the time of scans was classified as progressive, non-progressive (i.e. stable disease or regression) or mixed (i.e. both components with progression and components with regression). The classification was based on the combined conclusion of the clinical readings of MRI performed at the time of the study and the non-intervention [^18^F]FET PET scan when comparing to last prior imaging.

### Blood sampling and amino acid analysis

Blood samples for analysis of fasting amino acid concentrations (t = 0) were drawn on each scan day. In the PC group samples were also drawn prior to administration of the tracer (t = 60 min after protein consumption). To assess temporal dynamic of amino acid concentrations following protein consumption, additional samples were drawn prior (at t = 30) and after imaging (at t = 100) in five patients. Venous blood samples were collected in vacuum tubes containing EDTA, and a minimum of 0.5 ml of plasma was subsequently separated from each sample and stored at − 80 °C until analysis.

The sum of non-protein bound plasma levels of the LAT1-relevant AMAs (TYR, LEU, ILE, VAL, PHE, TRP, MET and HIS) was quantified using the MassTrak Amino Acid Analysis (AAA) Solution Kit (Waters Corporation, USA), an Acquity UHPLC system with a C18 BEH column (1.7 µm;2.1 × 150 mm) and an integrated photodiode array (PDA) detector (operating at λ = 260 nm) (all from Waters Corporation, USA) [23] Samples were analysed continuously in a total of three rounds and concentrations were measured in µmol/l. Inter-assay coefficient of variation (CV%) for TYR, LEU, ILE, VAL, PHE, TRP, MET and HIS was as follows: < 20% for concentrations < 10 µM; < 10% for AA concentrations between 10 and 20 µM and < 5% for concentrations > 20 µM (in-house validation, data are available upon request).

### [^18^F]FET PET image acquisition and reconstruction

[^18^F]FET-PET scanning was performed on a Siemens PET/CT Biograph Truepoint 40 or 64 scanner with both scans in each patient performed on the same scanner. A 40 min dynamic acquisition of the brain was started at the time of the intravenous injection of an average activity of 210 MBq (SD ± 9 MBq) [^18^F]FET. Default random, scatter, and dead time correction and low dose CT-based attenuation correction was applied on each scan. Image reconstruction of the last 20 min (20–40 min post injection) was performed by OSEM 3D (4 iterations, 16 subsets) with a matrix size of 336 × 336 × 74 (0.8 × 0.8 × 3 mm voxel size) and filtered with a 5 mm FWHM Gaussian filter. From the full 40 min acquisition both a static 10–30 min image and a 22-frame dynamic sequence (6 × 10 s, 4 × 15 s, 2 × 30 s, 2 × 60 s, 2 × 150 s, and 6 × 300 s) were generated applying a 3.5 mm filter and a 168 × 168 matrix, but using otherwise identical parameters.

### Image analysis

The 20–40 min. [^18^F]FET PET images were co-registered to post-contrast T1-weighted or T2-weighted FLAIR MRI, which was acquired not more than two days before the first [^18^F]FET-PET scan. Image analysis was performed on a clinical workstation (Syngo-TrueD, Siemens Healthcare, Erlangen, Germany). A crescent shaped background region of interest (ROI), encompassing the activity above 70% of maximum, was delineated in healthy appearing cortex of 4 contiguous brain slices above the insula in the contralateral hemisphere [24]. The region was drawn on the baseline scan and then copied to the co-registered intervention scan to ensure identical background regions. The biological tumour volume (BTV) was auto-contoured in 3D, defining tumour tissue at a threshold of above 1.6 of the mean standard uptake value in the background ROI (SUVB) [25]. The SUV was calculated from the decay corrected PET image tissue radioactivity concentration divided by the injected dose of radioactivity per kilogram of the patient's body weight. In patients without active tumour tissue a 1.0 cm in diameter circular ROI was placed on the centre of the largest contrast enhancing lesion on T1 MRI or, in cases without contrast enhancement, in the centre of the hyperintense T2 FLAIR signal changes. The maximal and the mean tumour uptake normalized to background brain tissue (TBR_max_, TBR_mean_) were calculated from the maximal and mean tumour SUV (SUVT_max_, SUVT_mean_).

In metabolically active tumours (TBR_max_ > 1.6) an isocontour volume of interest was drawn around maximal FET uptake in the 10–30 min image and the region was then copied to the dynamic time series to extract time-activity curves (TAC). For each TAC the curve pattern was visually classified as increasing (steadily increasing), plateau (increase followed by plateau > 20–40 min) or decreasing (early peak < 20 min followed by decrease) as previously described [26].

### Statistics and statistical considerations

The median [^18^F]FET uptake and SUV values were determined. Changes between the two scans were for each metric calculated as:

$$Difference=Intervention-Baseline$$

$$Relative\; difference \left(\%\right)=\frac{100*(Intervention-Baseline)}{Baseline}\%$$

$$Variation \left( \% \right) = \frac{{100*\left| {Intervention - Baseline} \right|}}{{\raise.5ex\hbox{$\scriptstyle 1$}\kern-.1em/ \kern-.15em\lower.25ex\hbox{$\scriptstyle 2$} *\left( {Intervention + Baseline} \right)}}\%$$

Due to non-normal distribution of continuous parameters, group differences were investigated by Mann–Whitney U test and within-subject changes (between days and pre-/post protein intake) by Wilcoxon paired sign rank test. Test–retest repeatabilities were assessed by scatter and Bland–Altman plots. Bias was calculated as mean difference, and 95% upper and lower limits of agreement (LoA) as bias ± 1.96 SD. In order to take into account the skewed distribution of differences (in particular for BTV) and to avoid a lower LoA boundary below zero, the LoA’s of the relative changes were determined by first calculating the mean and standard deviation (SD) of the difference (d) of the log transformed values, a strategy similar to a prior study [19]:

$$\text{d}=\text{ ln}\left(Intervention\right)-\text{ln}\left(Baseline\right)=\text{ln}\left(\frac{Intervention}{Baseline}\right)$$

The LoA’s of percent change from baseline were then calculated as:

$$LoA=\left({e}^{bias\pm 1.96 \cdot SD} - 1\right)\cdot 100\%$$

The associations of change in SUV and tumour metrics with changes in LAT1 relevant AMA were assessed by simple linear regression.

A two-sided significance level of 0.05 was adopted. All statistical analyses were performed using STATA 15 (StataCorp LCC, College Station, Tx).

## Results

**Fig. 2:**
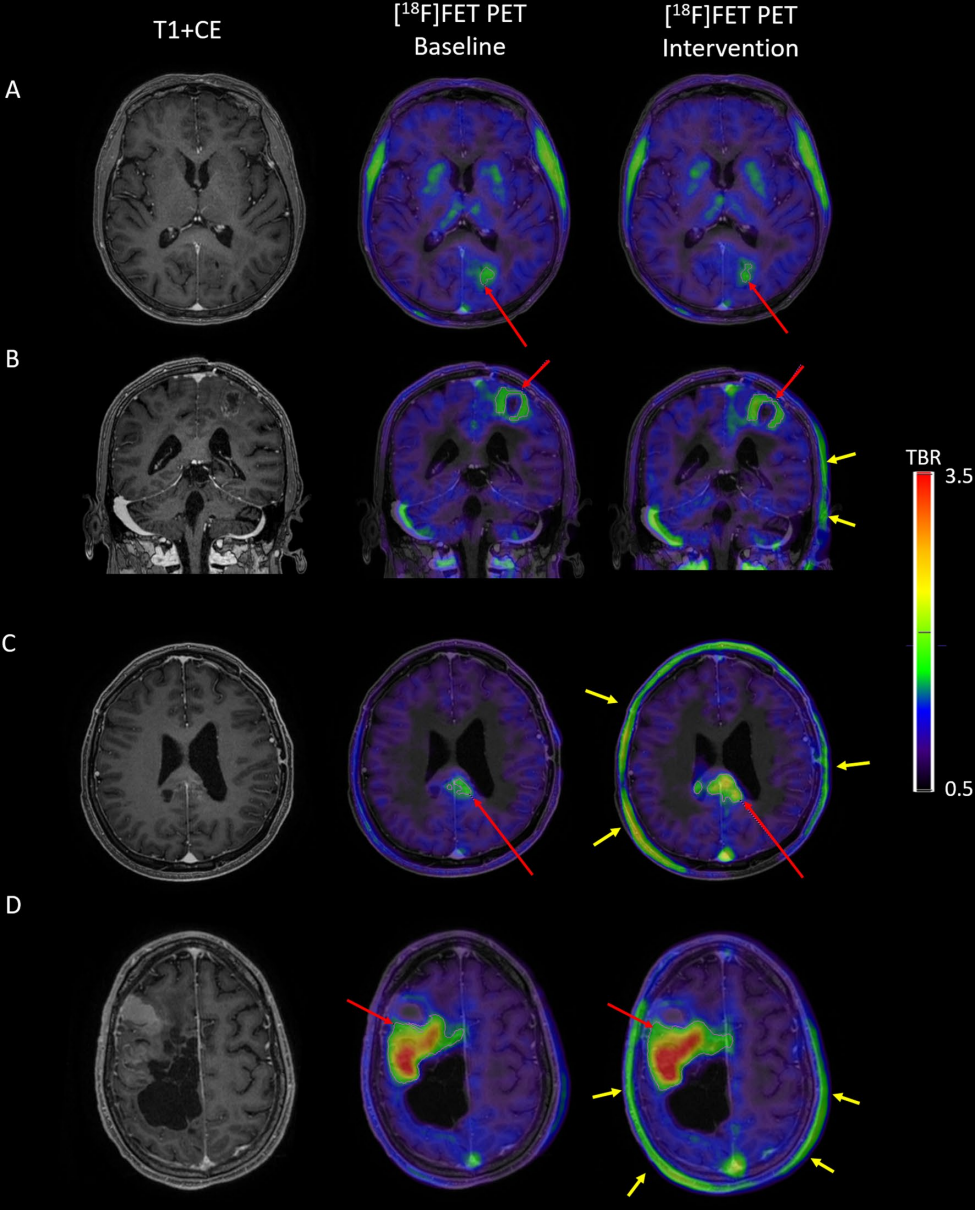
The influence of protein intake on [^18^F]FET PET uptake in tumour and extracerebral tissue. Left to right: post contrast T1 weighted MRI, baseline [^18^F]FET PET and intervention [^18^F]FET PET. Images are normalized to mean activity of a cortical background region. Repeat [^18^F]FET PET without oral protein intervention is shown in panel A and [^18^F]FET PET with and without oral protein intervention are shown in panel B-D. Note markedly relatively higher uptake in extracranial soft-tissue (yellow arrows), and also higher vascular activity in the post–protein intervention scans. **A** Patient #11, a 68-year-old female with oligodendroglioma, WHO grade 2. Baseline [^18^F]FET PET showed mild uptake in the left occipital region indicating active residual tumour with TBR_max_ of 1.75 and BTV of 1.06 ml. Seven days later a fasting intervention [^18^F]FET PET showed stable metrics (TBR_max_ 1.86 and BTV 0.66 ml). **B** Patient #2, a 65-year-old male with glioblastoma, WHO grade 4. Baseline [^18^F]FET PET showed mild-to-moderate uptake in left posterior frontal region with TBR_max_ of 1.99 and BTV of 8.0 ml. The protein intervention scan after consumption of 24 g of protein three days prior showed only slightly higher TBR_max_ of 2.18 and BTV of 9.2 ml. **C** Patient #1, a 56-year-old male with glioblastoma, WHO grade 4. Baseline [^18^F]FET PET showed mild uptake in splenium with TBR_max_ of 1.99 and BTV of 1.0 ml. The protein intervention scan after consumption of 48 g of protein three days later showed markedly higher TBR_max_ of 2.70 and BTV of 7.6 ml. **D** [^18^F]FET-PET with and without oral protein intervention: Patient #5, a 42-year-old male with astrocytoma, IDH mutant, WHO grade 3. [^18^F]FET PET showed uptake in the right frontoparietal region (TBR_max_ 4.56 and BT 34.4 ml). For the intervention scan seven days later scan 24 g of protein was consumed yielding an increase in TBR_max_ to 4.69 and BTV to 47.3 ml. The patient died two weeks after the intervention scan and increasing volume could reflect both protein-intake and tumour growth

Summary statistics of [^18^F]FET uptake, derived tumour metrics and amino acid measurements on each day are presented in Table 2. Detailed results from single participants are presented in supplementary data (Tables S1 and S2). There was no significant difference found comparing the baseline scans between the groups, making the groups comparable. Examples of repeated scans in the PC and the NP group are shown in Fig. 2.

**Table 2:** Tumour metrics

	NP group (n = 11)					PC group (n = 9)				
	Baseline	Intervention	Difference*	Variation (%)	Rel diff. (%)	Baseline	Intervention	Change	Variation (%)	Rel. diff (%)
*[*^*18*^*F]FET PET metrics*										
SUVB	0.92 [0.26]	0.96 [0.38]	0.00 [0.31]	16.8 [15.9]	0.2 [34.8]	1.06 [0.19]	0.74 [0.25]	− 0.28 [0.15]^†^	28.4 [28.4]	− 24.9 [21.2]
SUVT_mean_	1.53 [0.58]	1.43 [0.74]	0.02 [0.47]	12.0 [21.2]	2.4[29.9]	1.84 [0.76]	1.20 [0.36]	− 0.43 [0.38]^†^	25.8 [17.4]	− 22.9 [13.8]
SUVT_max_	1.78 [1.01]	2.06 [0.99]	0.04 [0.48]	10.2 [18.0]	2.2[22.2]	1.96 [0.95]	1.46 [0.65]	− 0.21 [0.38]^†^	15.0 [19.9]	− 14.0 [16.4]
TBR_mean_	1.72 [0.40]	1.75 [0.54]	0.00 [0.13]	4.3 [5.0]	0.0 [9.5]	1.66 [0.93]	1.68 [0.87]	0.04 [0.09]^§^	4.8 [5.4]	2.9 [6.8]
TBR_max_	1.95 [0.93]	2.17 [0.86]	0.06 [0.39]	4.5 [9.4]	0.0 [16.4]	1.78 [0.92]	1.90 [1.13]	0.11 [0.18]^§^	5.9 [5.7]	6.1 [10.2]
BTV [ml]	1.06 [3.7]	1.60 [4.10]	0.00 [1.44]	16.2 [34.3]	0.0 [37.1]	0.31 [3.60]	0.67 [7.60]	0.36 [2.80]^§^	31.6 [73.5]	15.0 [77.8]
*Total LAT1 relevant AMAs [µM]*										
Fasting	574 [100]	622 [48]	4 [76]	5.8 [6.9]	0.7 [14.1]	609 [95]	641 [100]	52 [31]^‡^	9.3 [5.4]	9.7 [5.8]
60 min	–	–	–	–	–	–	784 [220]	243 [178]^#^	31.6 [21.6]	37.5 [29.8]

Values are reported as median [QR]. *Intervention–baseline, P-value for intervention vs baseline ^§^*p* < 0. †*p* < 0.05 ‡*p* < 0.01; *p* values ^#^*p* < 0.01 for 60 min vs fasting

### Amino acid measurements

Blood samples from one patient (#17) failed to be collected prior to both scans. Detailed results of AMA plasma concentration measurements are presented in Supplementary Table S2. Median variation in fasting values was 6.1% (range 0.1–34.4%) in both groups pooled.

**Fig. 3:**
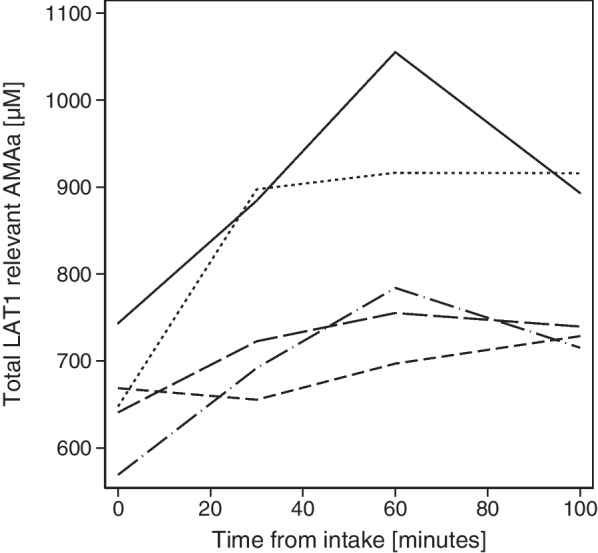
Plasma LAT1-relevant AMAs after protein intake. Sum of LAT1-relevant AMAs (TYR, LEU, ILE, VAL, PHE, TRP, MET and HIS) acid in five patients in the PC group measured at baseline (t = 0) and again after 30, 60 and 100 min

On the intervention day total LAT1 relevant AMA increased by a mean of 27% (absolute median increase 145μM, p = 0.008) from fasting values (t = 0) to time of tracer administration (t = 60) in the PC following protein consumption. In the five patients with repeated blood sampling plasma LAT1-relevant AMAs concentration was found to peak at 60 min after protein consumption (Fig. 3).

### Test-rest repeatability in NP group

**Fig. 4:**
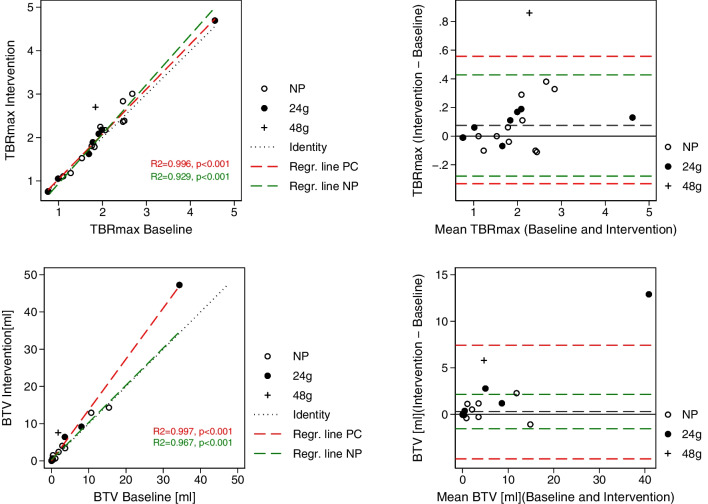
Test–retest repeatability. Scatter and Bland–Altman plots of TBR_max_ and BTV in the NP group (open circles). Observations from the PC group are shown superimposed (filed circles and + for single patient with double protein intake). In scatter plots dotted lines shows line of identity and coloured dashed lines show regression lines for the NP (green) and the 24g PC (red) groups. Dashed lines represent line of identity in scatter plot bias (black) and limits of agreement of the NP group (green) in Bland–Altman plots. Limits of agreement of groups combined are also shown (red)

**Fig. 5:**
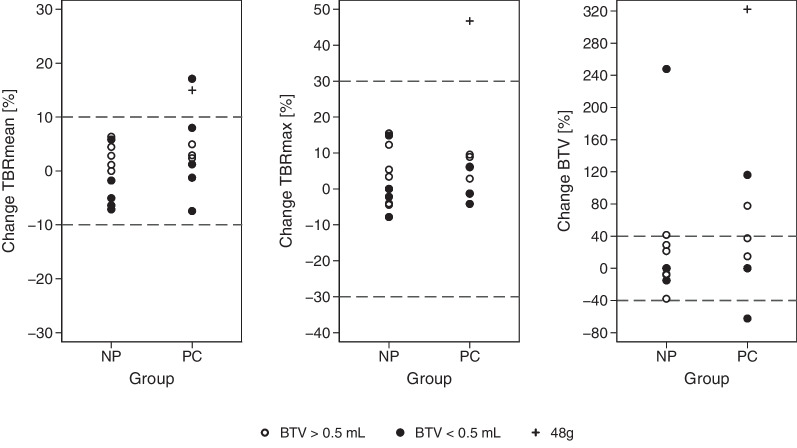
Relative change in [^18^F]FET PET metrics and PET-RANO criteria. Plots shows relative (%) change from baseline to intervention in patients with BTV above (hollow circles) and below (filled circles) PET RANO target lesion threshold in non-protein (NP) protein-consumption (PC) group. Dashed lines show PET RANO limits for stable disease

For variability and relative differences please refer to Table 2. There was no significant bias found in [^18^F]FET-tumour metrics between the baseline scan and the intervention scan in the NP group. Scatter and Bland–Altman plots are shown in Fig. 4, and relative changes are shown in Fig. 5. Upper and lower limits of agreement were 0.15 and − 0.13 for TBR_mean_, 0.43 and − 0.28 for TBR_max_, and 2.14 ml and − 1.53 ml for BTV (Table 3). Limits of agreement for the relative difference (ratio Intervention/Baseline) were wider for BTV than for TBR_mean_ and TBR_max_ indicating lower test-rest repeatability. Including also data from the PC group limits of agreement were wider with 0.20 and − 0.13 for TBR_mean_, 0.56 and − 0.33 for TBR_max_, and 7.43 ml and − 0.49 ml for BTV.

**Table 3:** Limits of agreement of clinical [^18^F]FET PET tumour metrics

	TBRmean		TBRmax		BTV (ml)		TTP (min)	
	NP	All	NP	All	NP	All	NP	All
*Difference. (intervention-baseline)*								
Bias	0.01	0.04	0.07	0.11	0.30	1.32	2.1	1.8
SD	0.07	0.08	0.18	0.23	0.94	3.12	3.9	4.8
Upper LoA	0.15	0.20	0.43	0.56	2.14	7.43	9.8	11.1
Lower LoA	− 0.13	− 0.13	− 0.28	− 0.33	− 1.54	− 4.79	− 5.6	− 7.6
*Relative difference (% change from baseline)*								
Bias^a^	0.00	0.02	0.03	0.05	0.16	0.25	0.09	0.05
SD^a^	0.05	0.06	0.08	0.10	0.51	0.64	0.16	0.21
Upper LoA ^b^ (%)	9.7	15.4	19.6	27.9	219.8	346.1	49.5	56.6
Lower LoA ^b^ (%)	− 9.0	− 9.9	− 11.8	− 14.2	− 57.3	− 63.0	− 19.5	− 30.0

*LoA* Limits of agreement, ^a^of ln(intervention/baseline), ^b^LoA calculated as (e^Bias±1.96*SD ^− 1)*100%

### Influence of protein intake and amino acid concentrations on tracer uptake

In the PC group a significant absolute and relative decrease was found in SUVB, SUVT_mean_ and SUVT_max_ on the intervention scan. A borderline significant increase in derived tumour metrics was also observed in the PC group (*p* = 0.06–0.08) which for TBR_max_ and BTV just reached statistical significance at the 0.05 level when including only patients with active tumour (*p* = 0.046 for both). For TBR_max_ one patient (#1, 48g protein) in the PC group the difference was outside limits of agreement determined from the NP group, and for BTV differences for three patients were outside limits of agreement (Fig. 4).

Background and tumour uptake tended to decrease with increasing LAT1 relevant AMAs (Suppl. Fig S1), and excluding a single outlying observation (#13) from the NP group with paradoxical large increase in both SUV values and LAT1 relevant AMAs, the negative associations of SUV with LAT1 relevant AMAs were highly significant for both SUVB (− 0.082 per 100 μM, *p* = 0.006), SUVT_mean_ (− 0.132 per 100 μM, p0.005) and SUVT_max_ (− 0.159 per 100 μM, *p* = 0.020). Increasing LAT1 relevant AMAs was associated with increasing BTV (0.98 ml per 100 μM, *p* = 0.043), but not with TBR_mean_ or TBR_max_.

### Time activity curves

There was no substantial change in curve patterns between scans, although classification changed in one case (#2 in PC group) from plateau/late decreasing to plateau and in another case (#15 in the NP group) from decreasing to plateau, both without change in time to peak). Limits of agreement for time to peak were relatively wide, but were identical in 9/14 of patients and differed by > 5 min only in two patients (both with increasing curve pattern).

## Discussion

This is the first controlled clinical prospective study to assess the influence of protein consumption on the test–retest variation of [^18^F]FET PET scans in gliomas. It is also the first study to report test–retest variability of standard [^18^F]FET-PET tumour metrics in humans. We found a borderline significant differences between the baseline scans and intervention scans in the PC group that with a few noticeable exceptions were within the variability observed in the NP group.

Knowing the fasting baseline test–retest repeatability and the impact of violation of fasting requirements in [^18^F]FET PET scans in gliomas is important when assessing a treatment response according to the recently proposed criteria for use in clinical trials, the PET RANO 1.0 criteria [27]. In this proposal, stable disease is defined as changes of TBR_max_ within 30%, TBR_mean_ within 10%, BTV within 40%, and no new measurable > 0.5 ml PET-positive lesions. The proposed cut-off levels were established based on results from clinical intervention trials, not test–retest studies, and are still in the process of validation. As shown in Table 4 and Fig. 5 relative changes in TBR_mean_ and TBR_max_ for patients with BTV > 0.5 ml were well within PET RANO criteria for stable disease [27], also in the PC group with the exception of the single patient consuming 48 g of protein. Test–retest repeatability in the NP group was good with median variation in TBR_max_ of 4.5% and in BTV of 16.2%. Compared to animal model study reporting test–retest repeatability of TBR_mean_ limits of agreement in animals tended to be narrower (− 0.1 and 0.1 vs. 0.13 and − 0.15 in our data), probably reflecting lower interindividual variability in the animal tumour model (also adjusting for tumour growth in the analysis). [28], To our knowledge only a single prior study has reported data from short-term (1 week) repeated [^18^F]FET PET scans in human gliomas. Ferjancic et al. reported SUV and BTV values of repeated [^18^F]FET PET scans in eight patients with newly diagnosed GBM performed 3–4 week after surgery and before radiotherapy [19]. From data presented by the authors limits of agreement of the ratio of scan 2 to scan 1 could be estimated to + 26% and − 21% for TBR_mean_, thus exceeding PET RANO criteria cut-off, while limits of agreement TBR_max_ ratio could be estimated to + 21% and − 18%, i.e. within PET RANO cut-off. The authors also reported considerable variability of BTV between the two scans with very wide limits of agreement for relative changes in BTV (+ 158% and − 64%), i.e. outside PET RANO criteria for stable disease.

**Table 4:** Limits of agreement of clinical [^18^F]FET PET tumour metrics in PET RANO measurable lesions (BTV > 0.05 ml)

	TBRmean		TBRmax		BTV (ml)		TTP (min)	
	NP	All	NP	All	NP	All	NP	All
*Difference. (intervention-baseline)*								
Bias	0.05	0.07	0.11	0.20	0.37	2.49	3.0	1.3
SD	0.05	0.08	0.21	0.28	1.4	4.16	4.5	4.7
Upper LoA	0.14	0.22	0.52	0.75	2.80	10.64	11.8	10.4
Lower LoA	− 0.05	− 0.08	− 0.30	− 0.34	− 2.05	− 5.66	− 5.8	− 7.9
*Relative difference (% change from baseline)*								
Bias^a^	0.02	0.04	0.04	0.09	0.09	0.26	0.13	0.03
SD^a^	0.03	0.04	0.08	0.16	0.16	0.50	0.18	0.22
Upper LoA^b^ (%)	7.6	12.6	21.7	49.5	49.5	249.3	61.1	58.0
Lower LoA^b^ (%)	− 2.5	− 4.1	− 10.4	− 19.5	− 19.5	− 51.6	− 19.6	− 33.3

*LoA* Limits of agreement, ^a^of ln(intervention/baseline), ^b^LoA calculated as (e^Bias±1.96*SD ^− 1)*100%

Using the same approach, limits of agreement for our data were + 10% and − 9% for TBR_mean_ and + 20 and − 12% for TBR_max_, thus indicating similar or slightly better repeatability. We also observed poorer repeatability of BTV measurements near or exceeding the PET RANO criteria, also in the NP group. Our study differs from that of Ferjancic et al. by including patients with smaller BTVs and by performing repeated scans at a later clinical stage where lower variability may be expected than in the immediate post-surgical period.

The two studies combined include a total of 18 patients (and only 14 if excluding the PC groups from the present study) with measurable disease according to PET RANO criteria. This sample is probably too small to reliably asses the test–retest variability and sensitive to the effects of single subject observations and methodological limitations. In particular the calculated limits of agreement may overestimate the variability. This underlines the paucity of such data that can serve to validate the proposed PET RANO criteria. Still, these data are to our knowledge the only human test–retest data available, and both studies suggest that BTV test–retest variability may exceed suggested BTV criteria for stable disease.

In general, large relative BTV changes are expected as even small changes in SUVB may significantly impact BTV, in particular in lesions that are small as in the present data and/or have diffuse “soft” borders. A measurable lesion of 0.5 ml corresponds roughly to a sphere with a diameter of 1 cm, meaning that diameter need only to change by 1 mm to 1.1 cm, i.e. by less than two voxels, to increase BTV by 40%. Accordingly, we found even larger relative changes in small tumours, but absolute change were in general < 2 ml in NP group. These observations suggest that even large relative changes in BTV should be interpreted with caution in small lesions, and possibly should be combined with an absolute volume change cut-off.

Protein intake decreased both tumour and background SUV, thus tending to outbalance the effects on derived tumour metrics, which were only borderline significant and to some extent depending on single observation rather than a general bias. Observations in the two patients with largest increases in LAT-relevant L-AMA (> 300 μM) were outside limits of agreement from the NP group. In patient #5 (Fig. 2D) BTV increased from 34 to 47 ml with no change in TBR_max_. This patient died 2 weeks after the intervention scan and the increase in BTV could thus reflect rapid tumour growth. In patient #1 (Fig. 2C) who received a dose of 48 g of oral protein the BTV as well as TBR_max_ and TBR_mean_ increased significantly. This patient was treated with bevacizumab for recurrent GBM and showing response to treatment on MRI. Although the patient did progress three months later, we find it unlikely that marked tumour growth should develop between two scans 3 days apart. It can be speculated that a sufficiently high protein load could increase tumour to background contrast using [^18^F]FET. However, the intake of such a very large load of protein is not likely to be relevant in a clinical setting. Based on the remaining patients consuming 24 g of protein, our data indicates that a moderate consumption of protein prior to [^18^F]FET-injection do not systematically suppress relative uptake in tumour tissue and that the [^18^F]FET-PET metrics are relatively robust. Still, variability tended to increase and the wider limits of agreement considering the two groups pooled may thus be considered the maximal variability in the case of patient non-compliance.

We also investigated repeatability of time activity curves obtained by 40 min dynamic imaging. Long frames and analysis of small tumour volumes causing TAC fluctuations may contribute to difficulties in TAC classification and determination of time-to-peak. Still, TAC curve patterns were classified differently in only two patients and time-to-peak differed by > 5 min also only in two patients with no clear association of oral protein with TAC pattern or time-to-peak.

We observed an association between the magnitude of the change in plasma LAT1-relevant AMAs at the injection time and the change in SUVs and [^18^F]FET tumour metrics. The reduction is likely caused by the competition of [^18^F]FET with plasma LAT1-relevant AMAs for transport into normal brain and gliomas. Serial blood sampling revealed variable plasma AMA increase after oral protein consumption, showing both slow gradual increase, plateau or decreasing concentration curves, probably reflecting differences in gastrointestinal absorption rate (Fig. 3, Suppl. Table S2). Bypassing the gastrointestinal tract with intravenous infusion of amino acids [13] would have ensured a stricter control of the amino acid plasma concentration for estimation of the dose–response relationship with [^18^F]FET uptake. A fully quantitative analysis with long dynamic acquisition with arterial measurements of plasma LAT1-relevant AMAs and [^18^F]FET would be needed to establish the relationship of AMA levels with tracer kinetics in more details. However, the aim was to assess the influence of non-intentional protein intake on tumour metrics and not to study the influence of amino acid levels on transport kinetics. The results are in accordance with a previous study using [^123^I]IMT SPECT under fasting conditions and a week later during intravenous infusion of a mixture of naturally-occurring LAT1-relevant AMAs in different brain tumours showing a reduction of uptake relative to blood of 46% in normal brain and of 53% in five glioma patients [15]. The decreased uptake in healthy appearing brain will give the appearance of relatively increased uptake in non-neural tissue with particular impact on blood activity and in skin (Fig. 2).

The guideline recommendation of a minimum 4 h prior fasting period is the best strategy to ensure stable metabolic conditions and should not be modified. However, clinical practice may necessitate the performance of a [^18^F]FET PET scan during suboptimal conditions. For non-compliant patients it may be useful to learn that prior moderate protein consumption does not necessarily have impact on clinical metrics, such as disappearance of active glioma tissue, and is associated with atypical imaging features, such as unusually high relative uptake in skin or in blood. Our results underline that suboptimal imaging conditions may be more common than expected in protocolled studies and routine clinical treatment monitoring alike. Although, all patients were given strict instruction to fast and testified to this prior to PET scanning, three NP patients (15%), #4, #13 and #20, all had differences in baseline to baseline total plasma AMA concentration above 100 µM. Emotional, cognitive, and social factors are important factors in adherence, and glioma patients may be more prone to nonadherence as a result of brain-damage [29]. Our data do suggest that varying levels of LAT1 relevant AMAs do influence imaging and that the recommendation of fasting prior to imaging should be maintained, but that non-compliance in a clinical setting may thus not necessitate rescheduling unless very large quantities of protein has been consumed. Some patients may have difficulties in fasting, e.g. children, and previous studies have permitted fasting for proteins thereby allowing children to eat food with very low amount of proteins such as fruits in the fasting period [30]. This is a pragmatic solution although the influence of fruit on [^18^F]FET uptake have not been investigated systematically.

This study has several limitations. A general challenge of test–retest studies in oncology is tumour growth in the interval between scans. Previously, the tumour volume median growth rate of untreated glioblastomas has been measured on contrast enhanced MRI to 1.4% pr. day [26] and the doubling time of glioblastoma may be as short as 10 days [27]. An animal study of implanted gliomas showed a 4% increase in [^18^F]FET TBR and tumour growth in just 48 h [28]. This indicates that our interscan interval of up to 1 week may have been too long, and we cannot rule out that tumour growth may have influenced observations in single patients. However, no such general time effect was observed, possibly because only patients between treatments were included. The participation in several scans within seven days may further have enhanced a selection bias for patients in a good performance status with low or no active tumour.

Finally, we included a limited sample size where only 20 of 29 recruited patients completed both scans thus further reducing statistical power. Also including a mixture of tumour types may reduce generalizability of the results. It would have been preferred to include a larger and more homogenous cohort including also more patients with larger tumour volumes. However, the result of the PET scan in such patients could require the change of therapy that should not be postponed in order to participate in the study, and would also introduce bias due to growth. These inevitable limitations underline the difficulties of performing test–retest studies in oncology imaging.

## Conclusions

The repeatability of [^18^F]FET PET scans was found good and reliable with TBR_mean_ and TBR_max_ showing less variability than BTV. Consuming 24 g of protein an hour before a [^18^F]FET PET scan decreases uptake of [^18^F]FET in both healthy appearing brain and tumour, but with no clinically significant impact on the most commonly used tumour metrics.

### Supplementary Information

Additional file1

## Data Availability

All data generated or analysed during this study are included in this published article and its supplementary information flies.

## Abbreviations

A2: Astrocytoma, IDH mutant, WHO CNS grade 2
A3: Astrocytoma, IDH mutant, WHO CNS grade 3
AMA: Amino acid
B: Activity in normal appearing cortex
BTV: Biological tumour volume
[^18^F]FET: O-(2-[^18^F]fluoroethyl)-l-tyrosine
GBM: Glioblastoma, IDH wild type, WHO CNS grade 4
HIS: Histidine
ICC: Intraclass correlation coefficient
ILE: Isoleucine
IQR: Interquartile range
[^123^I]IMT: L-3-[^123^I]iodo-alpha-methyl tyrosine
l-AMA: L-type amino acid
LAT: L-type amino acid exchanging transporter type 1
LEU: Leucine
MET: Methionine
MGMT: O^6^-methylguanin-DNA-methyltransferase
NP: Non protein group
OD2: Oligodendroglioma IDH mutant, 1p/19 codeleted, WHO CNS grade 2
OD3: Oligodendroglioma IDH mutant, 1p/19 codeleted, WHO CNS grade 3
PC: Protein consumption group
PET: Positron emission tomography
PHE: Phenylalanine
RANO: Response assessment in neurooncology
SUV: Standardized uptake value
SUVB: Standardized uptake value in healthy appearing background brain tissue
SUVT_max_: Standardized uptake value in the maximum tumour uptake
SUVT_mean_: Standardized uptake value in the mean tumour uptake
T_max_: Maximum tumour uptake
T_mean_: Mean tumour uptake
TBR_max_: Maximum tumour to background ratio
TBR_mean_: Maximum tumour to background ratio
TRP: Tryptophan
TTP: Time to peak
TYR: Tyrosine
VAL: Valine
