# Supplementary material for: A prospective clinical study of the influence of oral protein intake on [18F]FET-PET uptake and test–retest repeatability in glioma
Source: EJNMMI Res. 2024 Jun 26;14:58. doi: 10.1186/s13550-024-01119-0 (PMC11208353; doi:10.1186/s13550-024-01119-0)
Supplement: Supplementary file 1 — Additional file1 [file 13550_2024_1119_MOESM1_ESM.docx]

**Suppl. material**

**Suppl. Table S1. Static [^18^F]FET tumour uptake metrics and absolute change**

|  |  |  | First | Baseline | | | |  |  |  | Intervention | | | |  |  |  | Difference | | |
| --- | --- | --- | --- | --- | --- | --- | --- | --- | --- | --- | --- | --- | --- | --- | --- | --- | --- | --- | --- | --- |
| ID | Group | Protein | scan | SUVB | TBR_mean_ | TBR_max_ | BTV | TTP | Type |  | SUVB | TBR_mean_ | TBR_max_ | BTV | TTP | Type |  | TBR_mean_ | TBR_max_ | BTV |
| 1 | PC | 48 g | B | 1.06 | 1.67 | 1.84 | 1.8 | 17.5 | P/D |  | 0.66 | 1.92 | 2.70 | 7.6 | 17.5 | P/D |  | 0.25 (15.0%) | 0.86 (46.7%) | 5.8 (322%) |
| 2 | PC | 24 g + diet | I | 1.23 | 1.71 | 1.99 | 8.0 | 22.5 | P/D |  | 1.02 | 1.76 | 2.18 | 9.2 | 22.5 | D |  | 0.05 (2.9%) | 0.19 (9.5%) | 1.2 (15.0%) |
| 3 | PC | 24 g | B | 1.16 | 1.63 | 1.69 | 0.1 | 27.5 | I |  | 0.91 | 1.61 | 1.62 | 0.0 | 37.5 | I |  | -0.02 (-1.2%) | -0.07 (-4.1%) | -0.1 (-62.5%) |
| 4 | PC | 24 g + diet | I | 0.70 | 0.67 | 0.77 | 0.0 |  |  |  | 0.62 | 0.62 | 0.76 | 0.0 |  |  |  | -0.05 (-7.5%) | -0.01 (-1.3%) | 0.0 (0%) |
| 5 | PC | 24 g | B | 0.83 | 2.23 | 4.56 | 34.4 | 12.5 | D |  | 0.47 | 2.34 | 4.69 | 47.3 | 8.75 | D |  | 0.11 (4.9%) | 0.13 (2.9%) | 12.9 (37.5 %) |
| 6 | PC | 24 g | B | 1.21 | 1.66 | 1.78 | 0.3 |  |  |  | 0.71 | 1.68 | 1.89 | 0.7 |  |  |  | 0.02 (1.2%) | 0.11 (6.2%) | 0.4 (116%) |
| 7 | PC | 24 g | B | 1.20 | 1.69 | 1.91 | 3.6 | 32.5 | I |  | 0.90 | 1.73 | 2.08 | 6.4 | 37.5 | I |  | 0.04 (2.4%) | 0.17 (8.9%) | 2.8 (77.8%) |
| 8 | PC | 24 g | B | 1.05 | 0.50 | 0.76 | 0.0 |  |  |  | 1.13 | 0.54 | 0.75 | 0.0 |  |  |  | 0.04 (8.0%) | -0.01 (-1.3%) | 0.0 (0%) |
| 9 | PC | 24 g | B | 1.01 | 0.76 | 0.99 | 0.0 |  |  |  | 0.74 | 0.89 | 1.05 | 0.0 |  |  |  | 0.13 (17.1 %) | 0.06 (6.1%) | 0.0 (0%) |
| 10 | NP | - | B | 1.41 | 0.99 | 1.28 | 0.0 |  |  |  | 1.18 | 0.94 | 1.18 | 0.0 |  |  |  | -0.05 (-5.1%) | -0.10 (-7.8%) | 0.0 (0%) |
| 11 | NP | - | B | 0.96 | 1.66 | 1.75 | 1.1 | 32.5 | I |  | 1.14 | 1.66 | 1.81 | 0.7 | 32.5 | I |  | 0.00 ( 0%) | 0.06 (3.4%) | -0.4 (-37.7 %) |
| 12 | NP | - | B | 1.38 | 1.69 | 1.82 | 0.4 | 37.5 | I |  | 1.38 | 1.66 | 1.78 | 0.3 | 37.5 | I |  | -0.03 (-1.8%) | -0.04 (-2.2%) | -0.1 (-15.0%) |
| 13 | NP | - | B | 0.37 | 1.80 | 2.46 | 2.9 | 27.5 | I |  | 0.76 | 1.88 | 2.84 | 4.1 | 37.5 | I |  | 0.08 (4.4 %) | 0.38 (15.4%) | 1.2 (41.4%) |
| 14 | NP | -- | B | 0.85 | 1.73 | 2.06 | 1.9 | 32.5 | I |  | 0.82 | 1.75 | 2.17 | 2.4 | 32.5 | I |  | 0.02 (1.2%) | 0.11 (5.3%) | 0.5 (29.0%) |
| 15 | NP | - | B | 0.92 | 1.72 | 1.95 | 0.5 | 12.5 | D |  | 0.67 | 1.82 | 2.24 | 1.6 | 12.5 | P |  | 0.10 (5-8%) | 0.29 (14.9%) | 1.1 (248%) |
| 16 | NP | - | B | 0.73 | 1.40 | 1.53 | 0.0 |  |  |  | 0.80 | 1.30 | 1.53 | 0.0 |  |  |  | -0.10 (-7.1%) | 0.00 (0%) | 0.0 (0%) |
| 17^a^ | NP | - | B | 0.96 | 1.79 | 2.46 | 15.4 |  |  |  | 0.96 | 1.84 | 2.36 | 14.3 |  |  |  | 0.05 (2.8%) | -0.10 (-4.1%) | -1.1 (7.1%) |
| 18 | NP | - | B | 1.08 | 0.94 | 1.11 | 0.0 |  |  |  | 1.12 | 0.88 | 1.11 | 0.0 |  |  |  | -0.06 (-6.4%) | 0.00 (0%) | 0.0 (0%) |
| 19 | NP | - | B | 0.85 | 1.80 | 2.50 | 3.7 | 12.5 | P/D |  | 1.03 | 1.80 | 2.39 | 3.4 | 17.5 | P/D |  | 0.00 (0%) | -0.11 (-4.4%) | -0.3 (-8.1%) |
| 20 | NP | - | B | 0.82 | 1.88 | 2.68 | 10.7 | 17.5 | P |  | 0.69 | 2.00 | 3.01 | 13.0 | 17.5 | P |  | 0.12(6.4%) | 0.33 (12.3%) | 2.3 (21.4%) |

. ^a^Patient baseline was a 20 minute static scan.

Abbreviations: B = baseline, I = intervention, BTV= biological tumour volume, TBR_max_ = maximum tumour uptake to brain ratio, TBR_mean_ = mean tumour uptake to brain ratio, SUVB = standard uptake value in background region of interest, PC = protein consumption group, NP non-protein consumption group. TTP =time to peak, Type=time activity curve type (I= increasing, P =plateau, D = decreasing) with two types indicated for borderline curve types.

**Suppl. Table S2. Total LAT1 relevant plasma amino acid concentrations**

| ID | Group | Baseline (μM) | Intervention (μM) |  |  |  |
| --- | --- | --- | --- | --- | --- | --- |
|  |  |  | t=0 | t=30 | t=60 | t=100 |
| 1 | PC | 657.2 | 743.3 | 884.3 | 1055.2 | 893.1 |
| 2 | PC | 609.4 | 668.7 | 655.4 | 696.9 | 728.5 |
| 3 | PC | 609.5 | 647.7 | 897.4 | 916.4 | 915.9 |
| 4 | PC | 689.6 | 803.7 |  | 948.2 |  |
| 5 | PC | 531.0 | 585.8 |  | 861.5 |  |
| 6 | PC | 541.0 | 569.0 | 691.8 | 784.0 | 715.2 |
| 7 | PC | 626.2 | 640.8 | 722.6 | 755.1 | 739.5 |
| 8 | PC | 449.0 | 449.4 |  | 518.9 |  |
| 9 | PC | 476.4 | 528.8 |  | 632.7 |  |
|  | *Mean (SD)* | *576.6 (81.8)* | *626.4 (108.0)* | *770.3 (112.7)* | *796.5 (167.4)* ‡ | *798.4 (97.5)* † |
|  |  |  |  |  |  |  |
| 10 | NP | 517.5 | 497.3 | . | . | . |
| 11 | NP | 580.3 | 613.5 | . | . | . |
| 12 | NP | 518.6 | 497.8 | . | . | . |
| 13 | NP | 567.1 | 803.1 | . | . | . |
| 14 | NP | 546.5 | 601.8 | . | . | . |
| 15 | NP | 565.5 | 630.4 | . | . | . |
| 16 | NP | 589.1 | 597.5 | . | . | . |
| 17 | NP |  |  | . | . | . |
| 18 | NP | 646.0 | 645.6 | . | . | . |
| 19 | NP | 687.0 | 646.9 | . | . | . |
| 20 | NP | 830.0 | 639.9 | . | . | . |
|  | *Mean (SD)* | *604.8 (95.1)* | *617.4 (85.8)* |  |  |  |

†p<0.05 and ‡p<0.01 vs t=0.

Abbreviations: PC = protein consumption group, NP = non-protein consumption group.


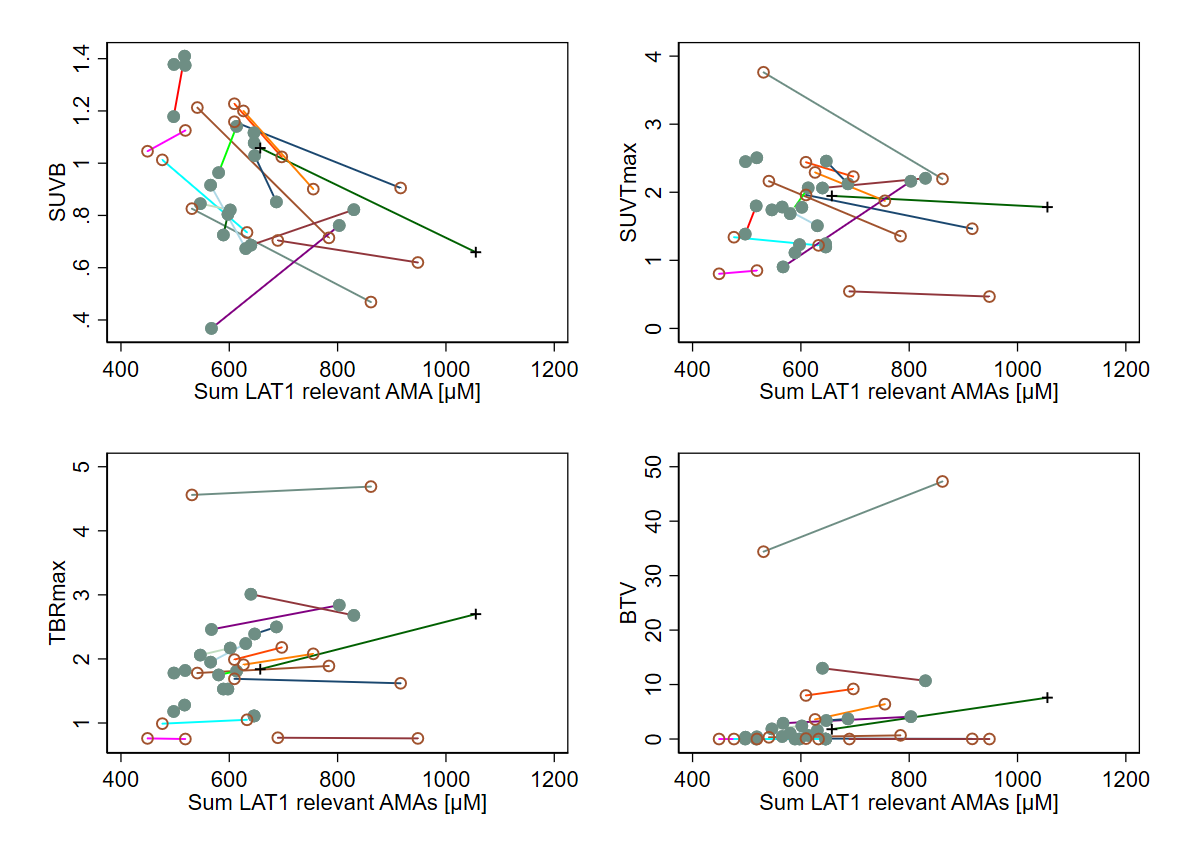


**Suppl. Fig S1. Association of pre-injection LAT1 relevant AMAs with SUV and tumour metrics**. Repeated measurements from single subjects from NP (solid circles) and PC group (hollow circles) are connected by coloured lines.
